# Visible-Light-Excited Room Temperature Phosphorescent Carbon Dots

**DOI:** 10.3390/nano10030464

**Published:** 2020-03-04

**Authors:** Sizhe Hu, Kai Jiang, Yuci Wang, Sui Wang, Zhongjun Li, Hengwei Lin

**Affiliations:** 1 School of Materials Science and Chemical Engineering, Ningbo University, Ningbo 315211, China; 2 Ningbo Institute of Materials Technology & Engineering (NIMTE), Chinese Academy of Sciences, Ningbo 315201, China; 3 College of Chemistry and Molecular Engineering, Zhengzhou University, Zhengzhou 450001, China; 4 International Joint Research Center for Photo-responsive Molecules and Materials, School of Chemical and Material Engineering, Jiangnan University, Wuxi 214122, China

**Keywords:** carbon dots, room temperature phosphorescence, visible-light-excitation, anti-counterfeiting

## Abstract

Carbon dots (CDs) with a room temperature phosphorescent (RTP) feature have attracted considerable interest in recent years due to their fundamental importance and promising applications. However, the reported matrix-free RTP CDs only show short-wavelength (green to yellow) emissions and have to be triggered by ultraviolet (UV) light (below 400 nm), limiting their applications in certain fields. Herein, visible-light-excited matrix-free RTP CDs (named AA-CDs) with a long-wavelength (orange) emission are reported for the first time. The AA-CDs can be facilely prepared via a microwave heating treatment of L-aspartic acid (AA) in the presence of ammonia and they emit unique orange RTP in the solid state with visible light (420 nm) excitation just being switched off. Through the studies of the carbonization process, the C=O and C=N containing moieties in the AA-CDs are confirmed to be responsible for the observed RTP emission. Finally, the applications of AA-CDs in information encryption and anti-counterfeiting were preliminarily demonstrated.

## 1. Introduction

Room temperature phosphorescent (RTP) materials have been continuously stimulating extensive research interests in recent years due to their significance in many fields of applications, such as optoelectronic devices, chemical sensing, bioimaging, anticounterfeiting, etc. [1,2,3,4,5,6,7,8]. The traditional pure-organic-small-molecule-based RTP materials, however, usually exhibit the shortcomings of complicated synthesis and purification, and require a highly crystallized form of compounds to produce RTP [2,4,9,10,11]. Moreover, most of the reported pure organic RTP materials have to be excited by ultraviolet (UV) light (below 400 nm), which significantly limits their range of applications (e.g., being not suitable for biology-relevant fields) [12]. In comparison with UV light, visible light shows less phototoxicity and deeper penetrability, which is more preferred for triggering RTP [13,14,15]. Therefore, it is still highly desirable to design and synthesize new classes of RTP materials with facile preparation process and visible-light excitable properties.

As a new type of luminescent nanomaterial, carbon dots (CDs) have received extensive attention due to numerous superior properties, such as easy preparation, low toxicity, high photostability, tunable emission, etc. [13,16,17,18,19,20,21,22,23,24]. Interestingly, RTP phenomena of CDs have been discovered recently by embedding or immobilizing in a variety of matrices, including poly (vinyl alcohol), polyurethane, layered double hydroxides, urea/biuret, zeolites and so on [25,26,27,28,29,30,31,32,33,34,35,36]. Although the introduction of matrices could realize RTP of CDs, the inherent chemical and physical properties of matrices would hinder applications of the RTP properties of CDs. More importantly, matrix-free RTP CDs have also been prepared very recently based on the concept of crosslink enhanced emission (CEE), self-immobilization fluorophores and specific elements (e.g., N, P and halogens) doping [37,38,39,40,41,42,43,44,45,46,47]. The reported matrix-free RTP CDs, however, only showed RTP emission colors from green to yellow and have to be excited by UV light as well [37,38,39,40,41,42,43,44,45,46,47]. To further expand the applications, it is significant to develop matrix-free RTP CDs holding long-wavelength (orange to red, and even to near infrared (NIR)) emissions and capable of being excited by visible-light, but this is still a formidable challenge.

**Figure 1:**
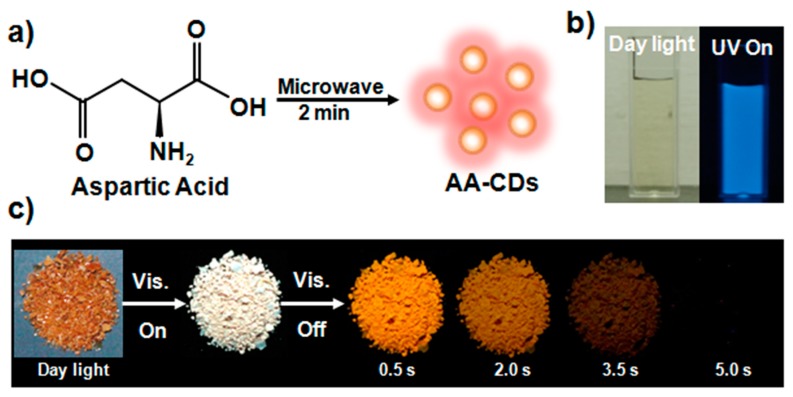
(**a**) Schematic illustration of the preparation process for visible-light-excited matrix-free room temperature phosphorescent (RTP)carbon dots (CDs) named AA-CDs. (**b**) Photographs of AA-CDs water dispersion under daylight and ultraviolet(UV) light (365 nm). (**c**) Photographs of the AA-CDs powder under the daylight, a visible light LED (420 nm) and delay time after ceasing the LED irradiation.

Based on the prerequisites for activating RTP in organic compounds and matrix-free CDs (e.g., introducing heteroatom to improve intersystem crossing (ISC) and producing matrix-like structures to self-immobilize fluorophores in CDs), [38,39,40,41] herein, a facile and quick method is reported to prepare visible-light-excited matrix-free RTP CDs (named AA-CDs) with orange emission. As shown in Figure 1a, L-aspartic acid (AA) was taken as the carbon precursor, and the AA-CDs can be obtained by microwave-irradiation heating AA in the presence of ammonia water (see Materials and Methods section for details). Notably, other kinds of natural amino acids do not produce RTP CDs by the same reaction conditions; this is because that AA could form crosslinked polymer-like structures under high temperatures and thus self-immobilize fluorophores in the AA-CDs [48,49]. A dilute aqueous dispersion of the purified AA-CDs exbibits common blue fluorescence (FL), like many other reported CDs, under the irradiation of a UV lamp (365 nm) (Figure 1b). Impressively, the solid powder of AA-CDs displays a warm white emission under a commercial blue-light LED (420 nm), and an orange afterglow is observed and lasts for several seconds to naked eye with the LED being just switched off (Figure 1c). To the best of our knowledge, this is the first example of achieving visible-light-excited matrix-free CDs with a long-wavelength (orange) afterglow.

## 2. Materials and Methods

### 2.1. Reagents

Reagent grade of L-aspartic acid (AA) was purchased from Aladdin Chemicals Co. Ltd. (Shanghai, China). An ammonia solution of superior purity (25–28%) was purchased from Sinopharm Chemical Reagent Co. Ltd. (Shanghai, China). All chemicals were used as received without further purification unless otherwise specified. Deionized (DI) water was used throughout this study.

### 2.2. Synthesis of AA-CDs and AA-CDs-2

Typically, 2.0 mL of ammonia solution was dissolved in 8 mL of DI water, and then 1500 mg of AA was slowly added into this solution with stirring, and the precursor was completely dissolved by ultrasonic treatment for 15 min. The as-formed homogeneous and transparent solution was transferred into a beaker and heated in a domestic microwave oven for about 2 min (750 W). After cooling to room temperature, the crude burnt yellow gel-like solid was obtained and could be completely dissolved by the addition of a sodium carbonate solution. For purifying the CDs, the above aqueous solution was firstly centrifuged (10,000 rpm/min for 20 min) and filtered through a 0.22μm membrane filter to remove large or agglomerated particles, then, the supernatant was collected and subjected to dialysis (MWCO: 1000 Da) for 3 days. Finally, the purified AA-CDs powder could be obtained by freeze-drying. Please note that the concentrations of AA and ammonia are critical for the successful preparation of AA-CDs. For example, solid-state white emissive CDs could be prepared by a similar procedure if lower concentrations of AA and ammonia were being used [48].

In order to form the further carbonized samples (AA-CDs-2), the crude, burnt, yellow gel-like solid was re-dissolved by the addition of DI water (8 mL) and ammonia solution (2 mL) and by then continuing to heat it in a domestic microwave oven for about 2 min (750 W). The above treatments were repeated three more times until a dark-brown gel-like solid was formed. The purifying procedure for the AA-CDs-2 was as the same as that of the AA-CDs, and the solid AA-CDs-2 powder could then be obtained by freeze-drying it.

### 2.3. Procedure

Preparation of the security ink: Typically, the AA-CDs aqueous dispersion (150 mg/mL) was used directly as security ink for anti-counterfeiting and information encryption.

Preparation of the interference fluorescent security ink (W-CDs): CDs with blue FL emission were prepared by a microwave assisted heating method. Typically, 0.5 mL of ethylenediamine was added into 20 mL of L-aspartic acid, aqueous dispersion (0.05 g/mL). Then, the mixture was heated in a domestic oven for 4 min (750 W). The excess precursors and resulting small molecules were removed by dialyzing against water through a dialysis membrane (MWCO: 1000 Da) for 3 days. The obtained CDs were dispersed in water (50 mg/mL) and employed as interference ink for information encryption.

### 2.4. Equipment and Characterization

Transmission electron microscopy (TEM) observations were performed on a Tecnai F20 microscope (FEI, Hillsboro, OR, USA). X-ray photoelectron spectroscopy (XPS) was carried out with ESCALAB 250Xi (Thermo Scientific, Waltham, MA, USA). Fourier transform infrared (FT-IR) spectra were obtained on a Nicolet 6700 FT-IR spectrometer (Thermo Nicolet Corp., Madison, WI, USA). Photoluminescence, phosphorescence emission and excitation spectra were measured on a Hitachi F-4600 spectrophotometer (Hitachi, Tokyo, Japan) at ambient conditions. UV-Vis absorption spectra were recorded on a PERSEE T10CS UV-Vis spectrophotometer (Persee, Beijing, China). FL and Phos lifetimes were measured using an EDINBURGH FLS 980photoluminescence spectrometer (Edinburgh Instruments, Wales, UK). Photographs of FL and Phos images were taken using a Canon camera (EOS 550, Tokyo, Japan) under a hand-held blue LED lamp (420 nm). PL QY measurements were carried out with a QE-2100 quantum efficiency measurement system (Otsuka Electronics, Tokyo, Japan).All the FL and RTP spectra and lifetimes were measured under aerobic atmosphere in this study unless otherwise noted.

## 3. Results and Discussion

### 3.1. Optical Properties of Carbon Dots (CDs)

**Figure 2:**
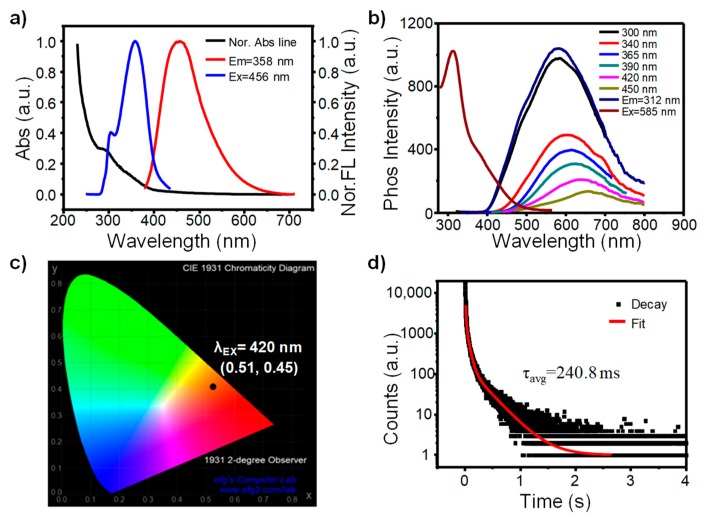
(**a**) The UV-Vis absorbance (black line), fluorescence (FL) emission (red line), and FL excitation (blue line) spectra of the AA-CDs water dispersion (1 mg/mL). (**b**) RTP emission spectra at different excitation wavelengths and excitation spectrum at the emission of 585 nm of the AA-CDs powder at ambient conditions. (**c**) CIE chromaticity coordinates of the AA-CDs powder under the excitation at 420 nm. (**d**) RTP decay spectrum and fitting curve (red line) of the AA-CDs powder at ambient conditions.

First of all, photophysical properties of the AA-CDs are investigated in detail. The aqueous dispersion of the AA-CDs exhibits an excitation-dependent FL feature with emission maxima at 456 nm under the optimal excitation wavelength of 358 nm (Figure 2a and Figure S1 in Supplementary Materials (SM)). The absolute FL quantum yield (QY) is measured to be 22.45% using an integrated sphere (Table S1 in SM). As shown in the UV-Vis absorption spectrum (Figure 2a), AA-CDs show a distinct absorption peak at 280 nm and a weak shoulder peak at about 340 nm, which can be attributed to the π→π* transition of C=C and n→π* transitions of C=O/C=N, respectively [31,50].The good consistency between their FL excitation and absorption spectra indicates that the C=O/C=N containing moieties in the AA-CDs should be responsible for the FL emission (Figure 2a). Moreover, it is found that the time-resolved FL spectrum of AA-CDs has to be fitted by a double-exponential function with an average lifetime of 9.99 ns (Figure S2 and Table S2 in SM). According to previous reports, the excitation-dependent FL emission and double-exponential decay features imply the existence of multiple emission centers within the AA-CDs [31,51,52,53]. In addition, the AA-CDs powder also exhibits an excitation-dependence FL feature (Figure S3 in SM) and the time-resolved spectrum has to be fitted by a double-exponential function as well. Both of the properties are the same as that of their aqueous dispersion, but with a shorter average lifetime of 8.04 ns (Figure S4 and Table S2 in SM). Impressively, an obvious orange RTP can be observed from the AA-CDs powder with visible light (420 nm) irradiation being just switched off (Figure 1c). Based on the RTP emission and excitation spectra (Figure 2b), the maximum RTP emission is located at 585 nm under the excitation wavelength of 312 nm, and meanwhile, the RTP emissions shift from 585 to 650 nm with the excitation wavelengths changing from 300 to 450 nm, indicating an excitation-dependence RTP property. Moreover, from Figure S5 (SM), it is clear to see that both FL and RTP exhibit two excitation bands at similar positions, although with different ratios, indicating that the RTP of the AA-CDs mainly arises from the same moieties as that FL. It is worthy to note here that the RTP of the AA-CDs is able to be excited by visible light, and this is very rare in pure organic RTP materials and CDs relevant systems [12]. Based on the RTP spectrum, the CIE 1931 chromaticity coordinates of AA-CDs RTP emission is determined to be (0.51,0.45) under the visible light (420 nm) irradiation (Figure 2c), which is located at the orange area and is in good consistence with the emission color (Figure 1c). To the best of our knowledge, this is the first example of visible-light excited matrix-free RTP CDs with a long wavelength (orange) emission.

To obtain a better understanding of the RTP properties, the RTP decay behaviors of AA-CDs powder were further examined. As shown in Figure 2d and Table S3 (SM), the RTP decay spectrum of AA-CDs powder has to be fitted with a tri-exponential function, indicating a multi-channel emissive nature. The three decay lifetime components were found to be 9.9, 51.78 and 298.76 ms (under excitation at 312 nm), respectively (Table S3 in SM), and an average lifetime was calculated to be 240.8 ms on the basis of the following equation [31,33,54].

$$\tau_{avg}=\sum \alpha_{i}\tau_{i}{}^{2}/\sum \alpha_{i}\tau_{i}$$

In addition, an afterglow emission from AA-CDs aqueous dispersion is observed at a low temperature (77 K) (Figure S6 in SM), but not at room temperature (even under nitrogen or argon inert atmospheres). This demonstrates that the RTP emission from AA-CDs powder should mainly arise from self-immobilization of the excited triplet species in their solid form [39,41,42]. Moreover, according to the low-temperature (77 K) FL and phosphorescence spectra of AA-CDs aqueous dispersion (Figure S6 in SM), an energy gap between the lowest T1 and S1 (Δ*E_ST_*) can be calculated to be 0.46 eV, and this small value of band gap is favorable for an effective ISC process to populate triplet excitations [33,55].

### 3.2. Morphologies and Structure Analyses of AA-CDs

**Figure 3:**
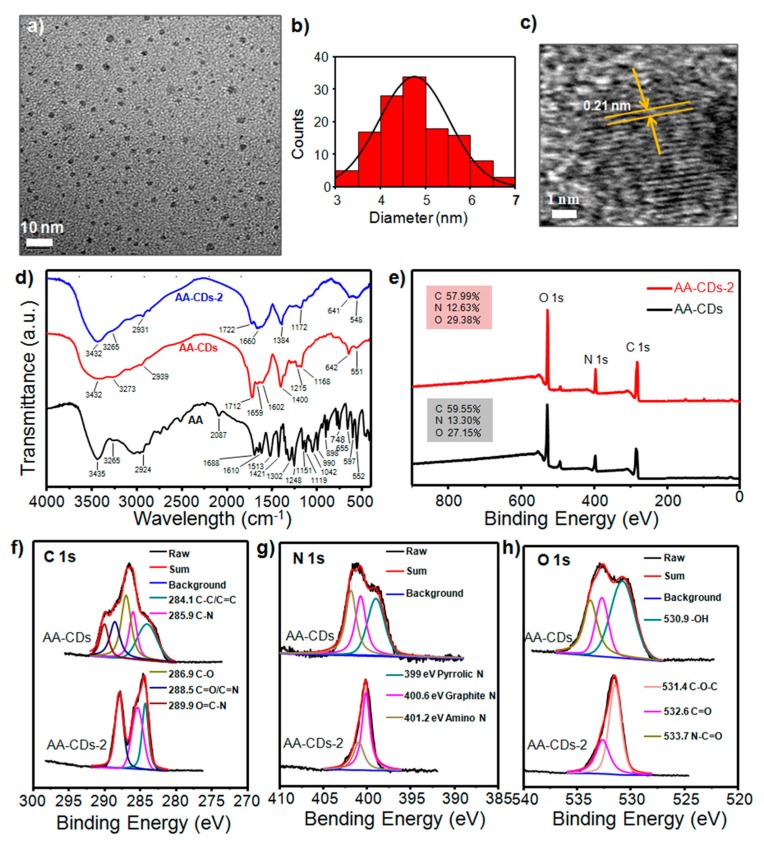
(**a**) Transmission electron microscopy (TEM) image, (**b**) the size histogram and (**c**) the high resolution TEM image of AA-CDs. (**d**) FT-IR spectra of L-aspartic acid (AA), AA-CDs and AA-CDs-2. (**e**)X-ray photoelectron spectroscopy (XPS) spectra of AA-CDs and AA-CDs-2. (**f**–**h**) High-resolution XPS and corresponding fitting results for the C 1s (**f**), N 1s (**g**), O 1s (**h**), spectra of AA-CDs and AA-CDs-2.

Subsequently, morphology and chemical structures of the AA-CDs were examined to provide deeper insights into their optical characteristics. First, transmission electron microscopy (TEM) measurements were performed to test the morphology of AA-CDs. As shown in Figure 3a,b, the TEM image displays that AA-CDs are quasi-spherical particles with an average size of 4.8 nm. The high resolution (HR) TEM observations confirm clear lattice fringes with a spacing of 0.21 nm (Figure 3c), which is in accordance with the (100) facets of graphite [56,57,58]. The surface groups and chemical compositions of the AA-CDs are followed and identified by Fourier transform infrared (FT-IR) and X-ray photoelectron spectroscopy (XPS). As seen in Figure 3d (red line), the FT-IR spectrum of AA-CDs shows obvious absorption peaks at 3432, 3273, 2939, 1712, 1659, 1400 and 1168 cm^−1^, which are attributed to the stretching vibrations of O–H, N–H, –CH2–, C=O, C=C, C–N and C–O bonds, respectively [41,48,49]. These FT-IR analyses could be further confirmed by the XPS characterizations. As shown in Figure 3e, the AA-CDs mainly contain C, N and O elements with corresponding contents to be 59.55%, 13.30% and 27.15%, respectively. More in detail, the HR XPS spectrum of C 1s indicates the presence of C–C/C=C (284.1 eV), C–N (285.9 eV), C–O (286.9 eV), C=N/C=O (288.5 eV) and N–C=O (289.9 eV) (Figure 3f) [49,59]. The HR XPS spectrum of N 1s contains three components that can be assigned to pyrrolic N (399.0 eV), graphite N (400.6 eV) and amino N (401.2 eV) (Figure 3g) [60,61]. The HR XPS of O 1s spectrum was deconvoluted into three peaks at 530.9 eV, 532.6 eV and 533.7 eV, corresponding to –OH, C=O and N–C=O, respectively (Figure 3h) [59,62].

### 3.3. Formation Process and Phosphorescence Mechanism of AA-CDs

In order to clarify the formation process and phosphorescence origins of the AA-CDs, the carbon precursor (i.e., AA) and a further carbonized sample (named AA-CDs-2, see Materials and Methods section for details of synthesis) were set up as references to investigated. From the FT-IR spectra (Figure 3d), the stretching vibrations of –OH bond at 3435 cm^−1^ and carboxyl group (COOH) at 1688 cm^-1^ decreased or almost disappeared, and accompanied by the emergence of carbonyl (amide C=O) stretching vibration at 1712 cm^−1^ from AA to the AA-CDs, indicating dehydration and amidation reactions occurred during the preparation process for the AA-CDs. From comparing the FT-IR spectra of AA-CDs and AA-CDs-2, one can observe the increased stretching vibration of C=C/C=N at 1660 cm^-1^ but decreased peak intensities at 3273 and 1712 cm^−1^, implying deeper carbonization and deamidation happened from AA-CDs to AA-CDs-2. These speculations are further verified by the XPS analyses. As shown in Figure 3e, AA-CDs and AA-CDs-2 consist of the same element compositions, but with different ratios (Table S4, SM). Moreover, the high resolution XPS spectra (Figure 3f–h) clearly demonstrated the increases of C–C/C=C, C–N and C=N/C=O, but decreases of C–O, N–C=O and O–H from AA-CDs to AA-CDs-2. The corresponding fitting results in Figure 3f–h are summarized in Table S5 (SM), which provide relatively quantitative alterations of the functional groups on AA-CDs and AA-CDs-2.

Then, the optical characteristics of AA and AA-CDs-2 were also investigated. As shown in Figure S7 (SM), the solid AA exhibits an excitation-independent FL character, while the AA-CDs-2 powder shows a similar excitation-dependence as that of the AA-CDs. Note that as the degree of carbonization increased from the AA-CDs to the AA-CDs-2, the FL excitation-dependence behavior became more obvious (Figures S1 and S8, SM). This demonstrates that with the carbonization of AA, more emissive centers are created, which should be responsible for the excitation-dependent FL character [59,63,64]. In addition, AA is found to emit very weak RTP in the solid state (too weak to be seen by naked eye) (Figure S9a, SM). The AA-CDs-2, however, exhibit similar RTP properties as that of the AA-CDs in the solid state (Figure S9b, SM), including the visible-light excitable feature (Figure S10, SM). The quantum yields (QYs) were found to decrease from the AA-CDs (22.45%) to the AA-CDs-2 (18.55%) (Table S1 in SM), implying some of the fluorophores on AA-CDs being consumed when they are subjected to further carbonization [47,63,64].

**Figure 4:**
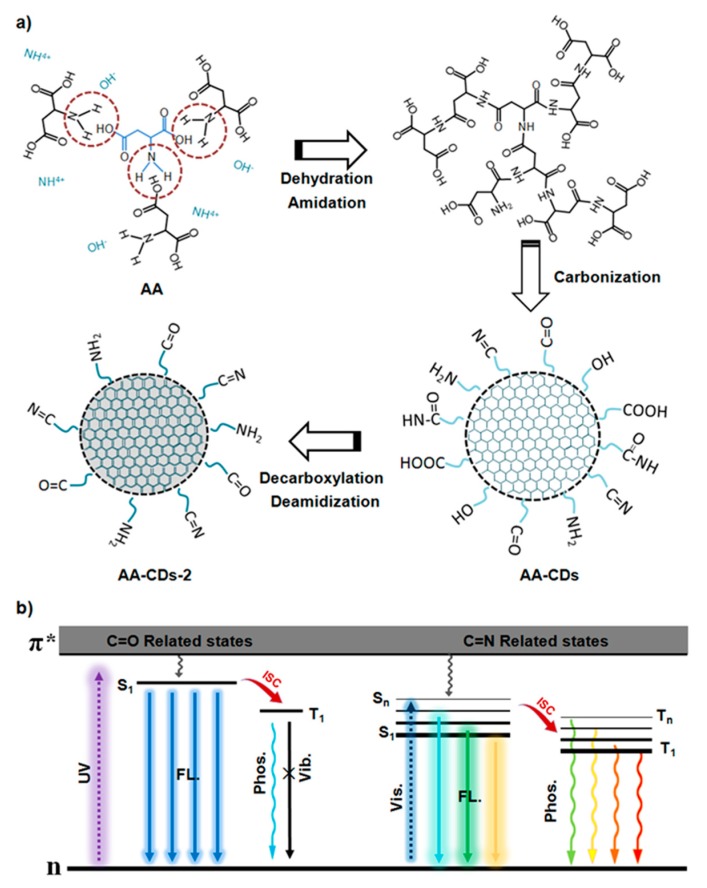
(**a**) A schematic illustration of the formation process from AA to the AA-CDs and AA-CDs-2. (**b**) Proposed FL and phosphorescence emission processes of the AA-CDs.

According to the above discussion, formation process and RTP origins of the AA-CDs could be tentatively proposed as follows: (i) Amide condensation, crosslinking polymerization and carbonization reactions could occur during the preparation process of the AA-CDs (Figure 4a); (ii) the crosslinked structure of the AA-CDs should be responsible for their enhanced FL in solution (CEE effects) and RTP in solid state (self-immobilization of emissive moieties) in comparison with AA; (iii) the more obvious excitation-wavelength dependence emission of the AA-CDs-2 than that of the AA-CDs could be ascribed to the presence of more C=N relevant fluorophores. The FL and RTP emission processes of the AA-CDs can be simply illustrated in Figure 4b.

### 3.4. Applications of CDs in Anti-Counterfeiting

**Figure 5:**
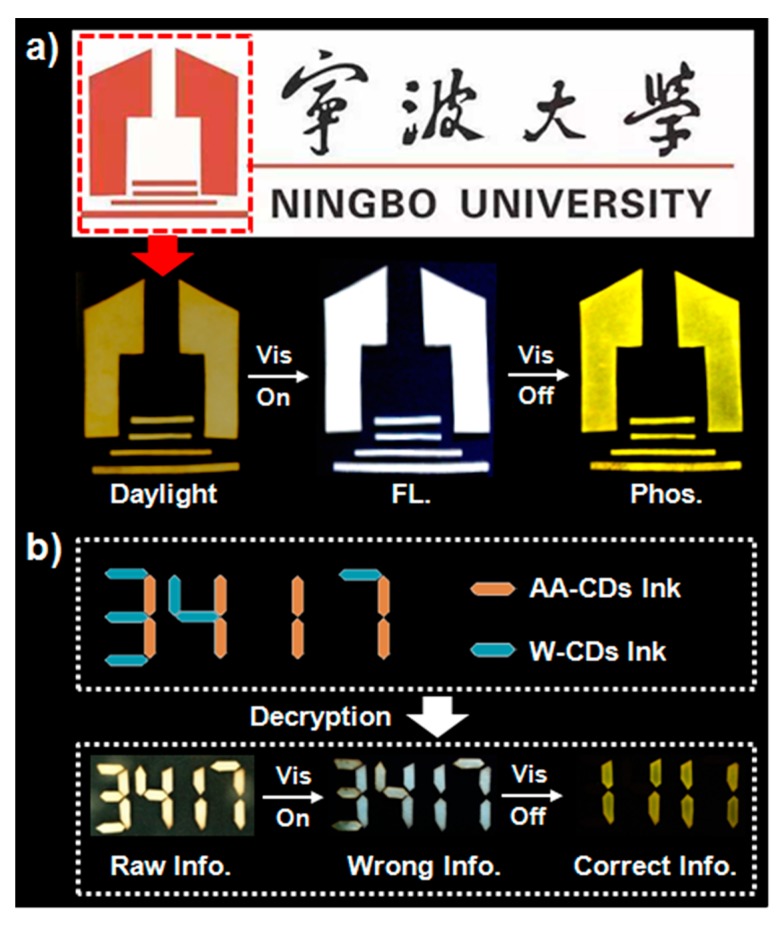
(**a**) Images of the badge of Ningbo University (made by filter paper that soaked in AA-CDs ink) under daylight, and visible light (420 nm) irradiation on and off, respectively. (**b**) Images of “3417” (made by filter paper that soaked in AA-CDs ink andinterference fluorescent security ink (W-CDs) ink) under daylight, and visible light (420 nm) irradiation on and off, respectively.

Thanks to the unique visible-light-excited RTP feature, the AA-CDs are considered to be more useful in certain fields of applications. As a preliminary example, their potentials in anti-counterfeiting and information decryption is demonstrated. To present such an application, the badge of Ningbo University (made by filter paper) was firstly soaked in the aqueous dispersion of AA-CDs and then thoroughly dried. As shown in Figure 5a, the badge of Ningbo University displays white emission and yellow RTP under a visible-light LED (420 nm) when it is “ON” and when it has just been turned off, respectively. For the information encryption and decryption applications of the AA-CDs, another kind of white emissive FL ink (named W-CDs, see Materials and Methods section for details of synthesis) was utilized as interference. As shown in Figure 5b, wrong information (i.e., “3417”) on the filter paper is observed under daylight and visible light (420 nm). The correct information (i.e., “1111”), however, can only be recognized under the phosphorescent mode, that is, taking the information with the visible light (420 nm) irradiation being just turned off.

## 4. Conclusions

In summary, the preparation of visible-light-excited matrix-free RTP CDs (i.e., AA-CDs) with orange emission feature is first reported in this study. The AA-CDs aqueous dispersion exhibits common bright blue FL emission and obviously excitation-dependent properties. The powder of AA-CDs, however, displays unique warm white emission and orange RTP under visible light (420 nm) irradiation being on and off, respectively. Further studies revealed that the crosslinked structure of the AA-CDs should be responsible for their specific optical properties. Finally, potential applications of the AA-CDs in the fields of anti-counterfeiting and information protection were preliminarily presented. More importantly, this study expanded the excitation wavelengths from the UV region to visible-light for matrix-free RTP CDs, and this will be very beneficial for applications in certain specific fields, such as biomedicine and optoelectronic devices, and such relevant works are now ongoing in our lab.

## Supplementary Materials

The following are available online at https://www.mdpi.com/2079-4991/10/3/464/s1, Figure S1: The FL emission spectra of the AA-CDs water dispersion under different excitation wavelengths and excitation spectrum at emission of 456 nm. Figure S2: Time-resolved FL spectrum and fitting curve of AA-CDs aqueous solution (λ_ex_ = 376 nm, λ_em_ = 456 nm) at ambient conditions. Figure S3: FL emission spectra of the AA-CDs powder under different excitation wavelengths and excitation spectrum at emission of 565 nm, Figure S4: Time-resolved FL spectrum and fitting curve of AA-CDs powder (λ_ex_ = 376 nm, λ_em_ = 565 nm) at ambient conditions, Figure S5: Normalized FL excitation spectrum of the AA-CDs water dispersion (1 mg/mL) and RTP excitation spectrum of AA-CDs powder in ambient conditions. Figure S6: The low temperature (77 K) FL and phosphorescence spectra of the AA-CDs water dispersion at air conditions under the excitation wavelength at 360 nm. Figure S7: (a) FL emission spectra at different excitation wavelengths and excitation spectra of AA powder, (b) FL emission spectra at different excitation wavelengths and excitation spectra of AA-CDs-2 powder. Figure S8: FL emission spectra at different excitation wavelengths and excitation spectrum at emission of wavelength 467 nm of AA-CDs-2 water dispersion. Figure S9: Phosphorescence emission spectra at different excitation wavelengths and excitation spectrum of the L-aspartic acid (a) and AA-CDs-2 (b) powders at ambient conditions. Figure S10: Photographs of the AA-CDs-2 powder at ambient conditions under day light, and visible (Vis.) light LED (λ_em_ = 420 nm) on and just being switched off, respectively. Table S1: QYs of AA-CDs and AA-CDs-2 aqueous dispersion. Table S2: Fitted parameters of the FL decay curves of the AA-CDs powder and water dispersion. Table S3: Fitted parameters of the phosphorescence decay curves of the L-aspartic acid, AA-CDs and AA-CDs-2 powder. Table S4: Relative contents of C, N and O elements of the AA-CDs and AA-CDs-2on the basis of the XPS data. Table S5: Relative contents of different functional groups in the AA-CDs and AA-CDs-2.
