# Supplementary material for: Visible-Light-Excited Room Temperature Phosphorescent Carbon Dots
Source: Nanomaterials (Basel). 2020 Mar 4;10(3):464. doi: 10.3390/nano10030464 (PMC7153588; doi:10.3390/nano10030464)
Supplement: Supplementary file 1 [file nanomaterials-10-00464-s001.pdf]

nanomaterials

Supplementary Material

Visible-Light-Excited Room Temperature Phosphorescent Carbon Dots

Sizhe Hu,[1,2] Kai Jiang,[2,*] Yuci Wang,[2,3] Sui Wang,[1] Zhongjun Li,[3] and Hengwei Lin[2,4,*]

[1] School of Materials Science and Chemical Engineering, Ningbo University, Ningbo 315211, China
[2] Ningbo Institute of Materials Technology & Engineering (NIMTE), Chinese Academy of Sciences, Ningbo 315201, China
[3] College of Chemistry and Molecular Engineering, Zhengzhou University, Zhengzhou 450001, China
[4] International Joint Research Center for Photo-responsive Molecules and Materials, School of Chemical and Material Engineering, Jiangnan University, Wuxi 214122, China
*Correspondence and requests for materials should be addressed to K. J. and H. L. (email: jiangkai@nimte.ac.cn, linhengwei@nimte.ac.cn).

Supporting Figures and Tables

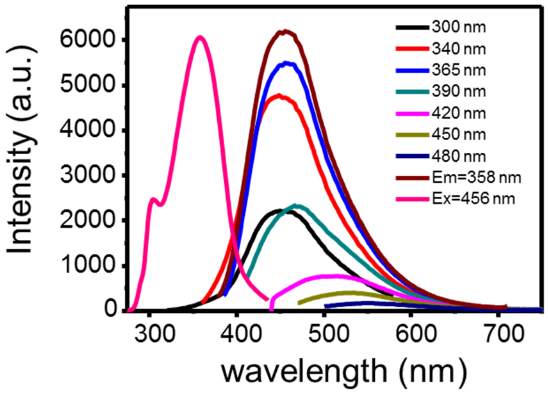

Figure S1. The FL emission spectra of the AA-CDs water dispersion under different excitation wavelengths and excitation spectrum at emission of 456 nm.

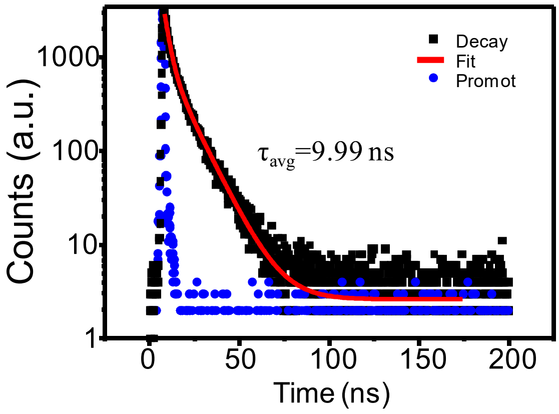

Figure S2. Time-resolved FL spectrum and fitting curve of AA-CDs aqueous solution (λ_{ex}=376 nm, λ_{em}=456 nm) at ambient conditions.

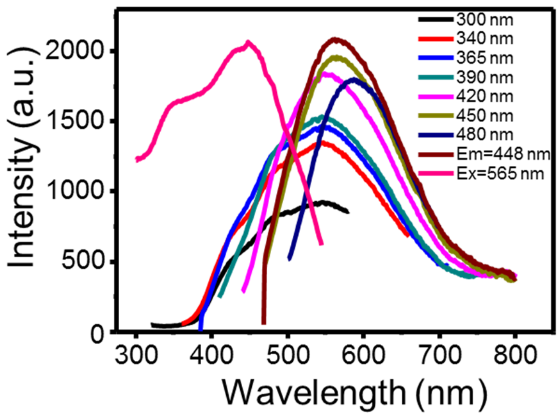

Figure S3. FL emission spectra of the AA-CDs powder under different excitation wavelengths and excitation spectrum at emission of 565 nm.

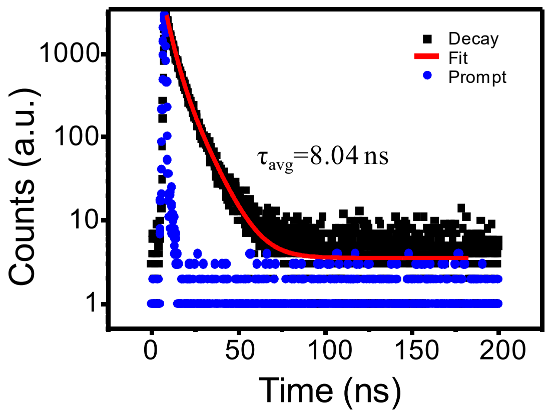

Figure S4. Time-resolved FL spectrum and fitting curve of AA-CDs powder (λ_{ex}=376 nm, λ_{em}=565 nm) at ambient conditions.

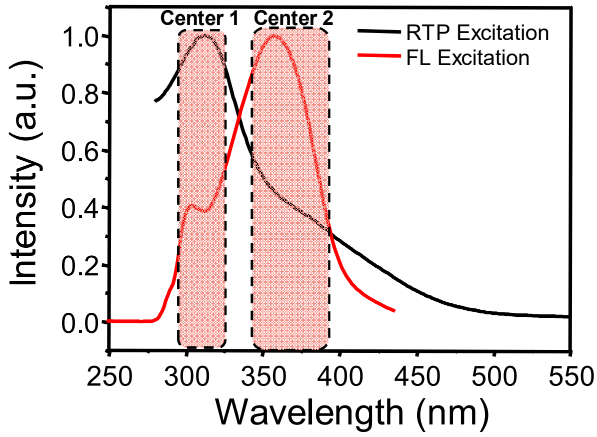

Figure S5. Normalized FL excitation spectrum of the AA-CDs water dispersion and RTP excitation spectrum of AA-CDs powder under ambient conditions.

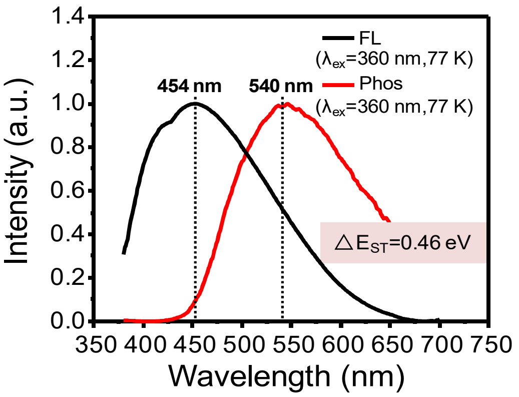

Figure S6. The low temperature (77 K) FL and phosphorescence spectra of the AA-CDs water dispersion at air conditions under the excitation wavelength at 360 nm.

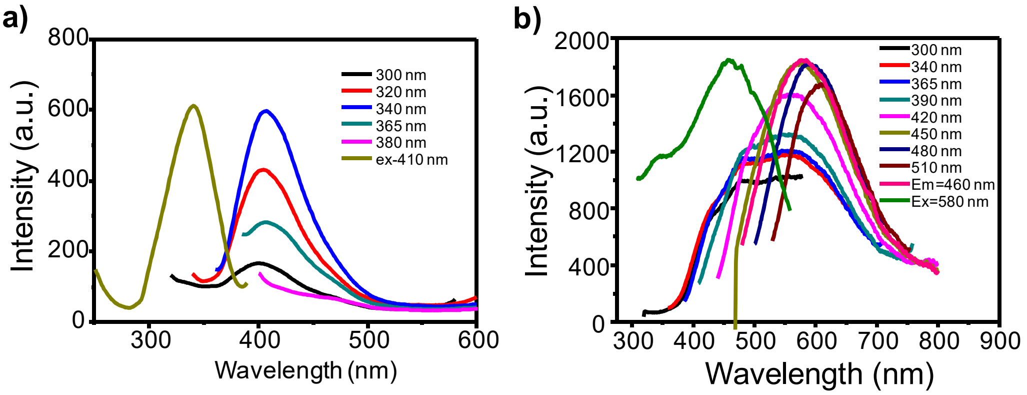

Figure S7. (a) FL emission spectra at different excitation wavelengths and excitation spectra of AA powder. (b) FL emission spectra at different excitation wavelengths and excitation spectra of AA-CDs-2 powder.

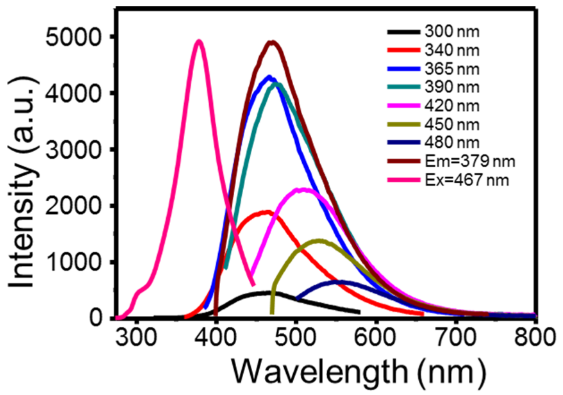

Figure S8. FL emission spectra at different excitation wavelengths and excitation spectrum at emission of wavelength 467 nm of AA-CDs-2 water dispersion.

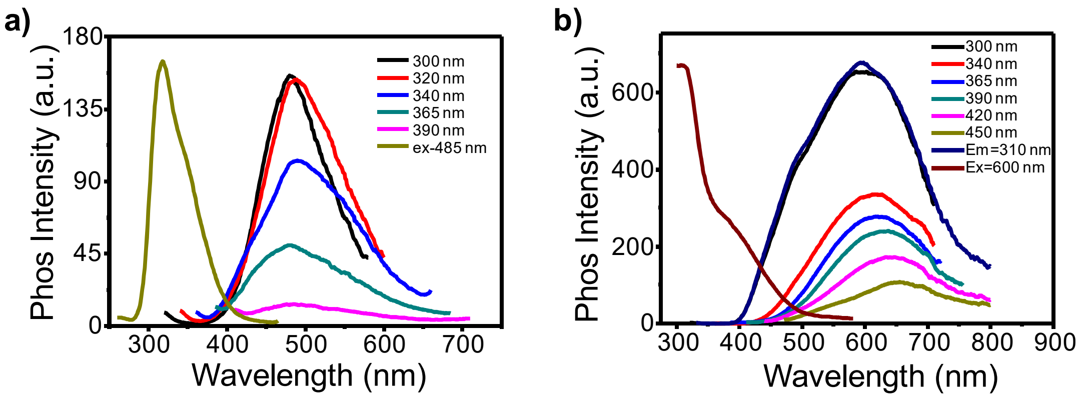

Figure S9. Phosphorescence emission spectra at different excitation wavelengths and excitation spectrum of the L-aspartic acid (a) and AA-CDs-2 (b) powders at ambient conditions.

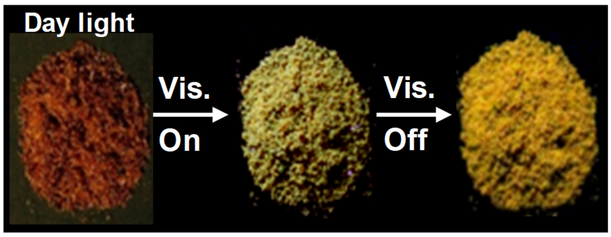

Figure S10. Photographs of the AA-CDs-2 powder at ambient conditions under day light, and visible (Vis.) light LED (λ_{em}=420 nm) on and just being switched off, respectively.

Table S1. QYs of AA-CDs and AA-CDs-2 aqueous dispersion.

Sample	Solvent	λ_{ex} (nm)	Φ_1 (%)	Φ_2 (%)	Φ_3 (%)	Φ_{avg} (%)	$\Phi_{corr.}$ (%)
**Rh-6G**	EtOH	488	95.92	94.36	95.09	95.12	95
**AA-CDs**	H_2O	365	22.30	22.54	22.62	22.48	22.45
**AA-CDs-2**	H_2O	365	18.26	18.76	18.79	18.57	18.55

Table S2. Fitted parameters of the FL decay curves of the AA-CDs powder and water dispersion.

Sample	Solvent	τ_1 (ns)	B_1 (%)	τ_2 (ns)	B_2 (%)	τ_{avg} (ns)	ϕ
**AA-CDs**	Powder	3.86	50.96	9.76	53.95	8.04	1.27
**AA-CDs**	H_2O	2.73	37.63	11.07	62.37	9.99	1.17

Table S3. Fitted parameters of the phosphorescence decay curves of the L-aspartic acid, AA-CDs and AA-CDs-2 powder.

Sample	τ_1 (ms)	B_1 (%)	τ_2 (ms)	B_2 (%)	τ_3 (ms)	B_3 (%)	τ_{avg} (ms)	ϕ
**AA**	5.37	15.76	33.86	51.10	192.53	33.13	157.09	1.27
**AA-CDs**	9.90	21.92	51.78	48.04	298.76	30.04	240.79	1.16
**AA-CDs-2**	2.82	9.40	31.85	64.93	168.61	25.68	123.89	1.11

Table S4. Relative contents of C, N and O elements of the AA-CDs and AA-CDs-2 on the basis of the XPS data.

Sample	C (%)	N (%)	O (%)
**AA-CDs**	59.55	13.3	27.15
**AA-CDs-2**	57.99	12.63	29.38

Table S5. Relative contents of different functional groups in the AA-CDs and AA-CDs-2.

Sample	C 1s					N 1s			O 1s			
	C-C/C=C	C-N	C-O	C=N/C=O	N-C=O	Pyrrolic N	Graphite N	Amino N	O-H	C-O-C	C=O	N-C=O
**AA-CDs**	24.02	17.02	29.17	17.44	12.34	36.77	30.26	32.91	48.53	-	22.40	29.08
**AA-CDs-2**	26.94	35.86	-	37.21	-	-	68.03	31.97	-	72.33	27.67	-
